# Sharing Roles in Home Care: Family Care Partners and Paid Caregivers

**DOI:** 10.1002/alz.091375

**Published:** 2025-01-09

**Authors:** Chanee D Fabius, Joseph J Gallo, Jennifer L Wolff

**Affiliations:** ^1^ Johns Hopkins Bloomberg School of Public Health, Baltimore, MD USA

## Abstract

**Background:**

Little is known about the prevalence and nature of role‐sharing among family care partners and paid caregivers.

**Method:**

We studied 440 participants in the 2015 National Health and Aging Trends Study (NHATS) receiving paid help with self‐care, mobility, or medical care. Guided by Litwak’s task specificity model, we focus on frequency of receiving paid help only, help from care partners only, and role‐sharing by task. We examine whether role sharing and reliance on paid help only differs by older adults’ Medicaid‐enrollment and dementia status.

**Result:**

Older adults whose helping arrangements did (versus did not) involve role‐sharing were more often living with dementia (48.7% vs. 25.6%, *p*<0.001) but less often Medicaid‐enrolled (32.1% vs. 49.4%, *p*<0.01). Those living with (versus without) dementia more often experienced role‐sharing in eating (OR 3.9 [95% CI 1.20, 8.50]), bathing (OR 2.7, [95% CI 1.50, 4.96]), dressing (OR 2.1 [95% CI 1.14, 3.86]), toileting (OR 2.9 [95% CI 1.23, 6.74]), and indoor mobility (OR 2.8 [95% CI 1.42, 5.56]), and less often received help solely from paid helpers with medication administration (OR 0.24, [95% CI 0.12, 0.46]). Those who were (versus not) enrolled in Medicaid more often received paid help only in dressing (OR 2.0 [95% CI 1.12, 3.74]), getting around outside (OR 2.4 [95% CI 1.28, 4.36]), and inside (OR 4.3 [95% CI 2.41, 7.62]), as well as with doctor visits (OR 2.8 [95% CI 1.29, 5.94]).

**Conclusion:**

Findings highlight heterogeneity and the high prevalence of role‐sharing in paid care, particularly for older adults living with dementia and not enrolled in Medicaid. Findings lay the foundation for interventions that align with national recommendations to increase understanding of and strengthen relationships between family caregivers and paid caregivers.

